# A Personal Reflection on the Late Rinaldo Bellomo, One of the Most Prolific Researchers in Intensive Care Medicine

**DOI:** 10.1111/aas.70097

**Published:** 2025-07-10

**Authors:** Markus B. Skrifvars

**Affiliations:** ^1^ Department of Anaesthesiology and Intensive Care Medicine Helsinki University Hospital and University of Helsinki Helsinki Finland

The intensive care community was deeply saddened by the sudden and premature death of Professor Rinaldo Bellomo at the age of 67. His passing marks the loss of one of the most prolific and influential researchers in the history of intensive care medicine. After emigrating from Italy to Australia in the 1990s, Rinaldo became one of the founding members of the Australia and New Zealand Intensive Care Society Clinical Trials Group (ANZICS CTG). Under his leadership and inspiration, the group produced a vast array of high‐quality studies that have shaped the modern care of critically ill patients [1]. His research interests were impressively broad, encompassing acute kidney injury [2], sepsis [3], post‐cardiac arrest care [4], fluid therapy [5], vasopressor use [6], nutrition [7], medical emergency teams [8], mechanical ventilation, delirium, traumatic brain injury [9], COVID‐19 [10], and blood transfusion [11]. He contributed to all forms of research—basic science, small observational studies, large registries, pilot investigations, and large multicenter phase III trials—with the same passion and intellectual rigor.

What set Rinaldo apart was not just his productivity, but the consistency and humility with which he engaged in the research process. Whether co‐authoring a manuscript for a leading journal or a more modest submission, he devoted the same energy to improving the clarity, precision, and scientific value of the work. It did not matter whether his collaborators were senior investigators or early‐career students—he was equally generous with his time and mentorship. He was certainly not the kind of senior author who expected credit without contribution; quite the contrary.

Rinaldo's determination to pursue important questions—often long after others had moved on—was remarkable. A case in point is the evolving and controversial topic of vitamin C therapy in sepsis. After an early, small, before‐and‐after study suggested improved survival with intravenous vitamin C and thiamine in septic shock, the field rapidly filled with both enthusiasm and skepticism [12]. Rinaldo led one of the initial randomized controlled trials in this area [13]. When the subsequent LOVIT study suggested a potential signal of harm [14], many researchers considered the matter closed. But not Rinaldo. Instead, he explored a critical nuance often overlooked—the biochemical differences between vitamin C formulations, specifically ascorbic acid and ascorbate. His team pursued further investigations, including a large‐animal (sheep) study [15] and a phase I clinical trial assessing mega‐dose vitamin C [16]. Whether these differences in compound or dose will ultimately prove to be clinically meaningful remains to be seen [17]. However, Rinaldo's persistence exemplified his scientific ethos: to keep asking questions, and to keep testing hypotheses until the evidence was clear.

He was also a gifted and tireless speaker. At the International Symposium on Intensive Care and Emergency Medicine (ISICEM), where most faculty deliver five to six lectures, Rinaldo often gave 10–15, maintaining the same energy and insight throughout. His presentation style was elegant and unpretentious—just a few simple slides, and a torrent of thoughtful, evidence‐based commentary. He welcomed difficult questions and enjoyed challenging dogma. Despite his Italian heritage and a passion for debate, he never lost his composure. Even in heated discussions, he remained calm, articulate, and respectful, always grounding his arguments in science.

Many Scandinavian researchers have benefited directly from his mentorship. Several spent extended periods in Melbourne and later brought his collaborative and rigorous approach back to Scandinavia [18, 19, 20]. In recognition of this lasting connection with Finnish intensive care researchers, Rinaldo was awarded an Honorary Doctorate by the University of Helsinki in June 2022. His honorary lecture addressed the physiology and potential of angiotensin II as a vasopressor—an area in need of innovation after years of stagnation in the pharmacologic management of shock [6].

Rinaldo was, in every sense, a renaissance man. His interests extended beyond medicine into history, philosophy, literature, and politics. He had the rare ability to draw connections across disciplines and to raise thoughtful, sometimes challenging, questions that others had not even considered. On a personal note—and although it may sound naïve and even exaggerated—I often told myself, “I will never meet Leonardo da Vinci, but I have met Rinaldo Bellomo, and that must be almost as good.”

He approached even serious work with a touch of dark humor and never took offense when his theories were challenged. One quote I will never forget: “When you start to believe your own research bullshit, it's time to retire.” Sadly, Rinaldo did not live to retire from clinical practice nor research. But I often wonder if he ever would have retired from his research? He was endlessly curious and brimming with new ideas. And given his willingness to question even his own work, perhaps—by his own criteria—he would have never even needed to retire.

For all of us working in intensive care research, the field feels markedly emptier without Rinaldo's guidance and help. But I am certain he would urge us to continue—to challenge assumptions, ask better scientific questions, and test every theory rigorously and aim for large international pragmatic trials [21, 22, 23, 24, 25]. We will definitely try to do so but it will not be as enjoyable, nor as inspiring, without Rinaldo's voice in the conversation.

Use of AI: I declare that I have written this paper myself without the use of artificial intelligence. However, after completing the first draft I have used CHATGPT on June 20, 2025 in order to improve the quality of the language. I have reviewed and modified the version created by CHATGPT.

## Author Contributions

Markus B. Skrifvars is the only author of this article i.e., Markus B. Skrifvars wrote this editorial by himself.

## Conflicts of Interest

The author declares no conflicts of interest.

## Data Availability

Data sharing not applicable to this article as no datasets were generated or analysed during the current study.
